# Supplementary material for: SMRT and NCoR1 fine-tune inflammatory versus tolerogenic balance in dendritic cells by differentially regulating STAT3 signaling
Source: Front Immunol. 2022 Sep 27;13:910705. doi: 10.3389/fimmu.2022.910705 (PMC9552960; doi:10.3389/fimmu.2022.910705)
Supplement: Supplementary file 7 [file DataSheet_1.pdf]

Supplementary Figure 1

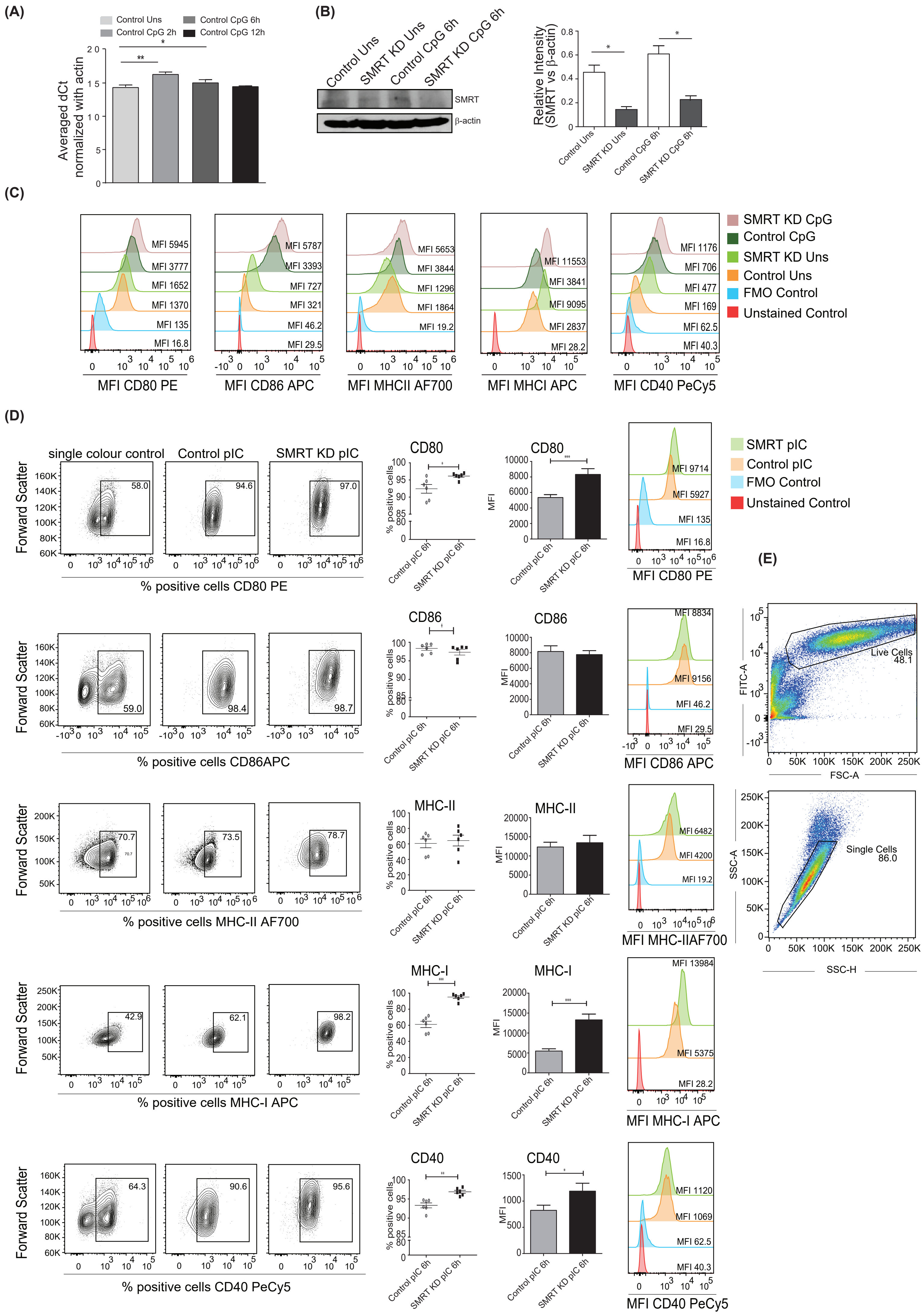

Figure S1. SMRT depleted cDC1 DCs exhibit enhanced co-stimulation upon TLR9 and TLR3 stimulation. (A) RT-qPCR showing the relative transcript expression of Ncor2 (averaged dCt normalized with β-actin) in unstimulated and 2h, 6h, and 12h CpG stimulated cDC1 (n=3). **(B)** Western blot showing SMRT protein expression in unstimulated and 6h CpG stimulated control and SMRT KD cDC1. Densitometry analysis depicting intensity of SMRT bands normalized with β-actin used as loading control (n=3). **(C)** Flow cytometry analysis depicting histogram with MFI shifts of co-stimulatory molecules CD80, CD86, CD40 and activation marker MHC-II, and MHC-I in unstimulated and 6h CpG stimulated control and SMRT KD cDC1 compared to their unstained and respective FMO control. **(D)** Contour plots depicting percent postive cells and MFI of co-stimulatory molecules and activation markers in 6h pIC stimulated control and SMRT KD cDC1 along with their respective unstained, single colour and FMO control. For staining MHC-I we did not use any other fluorochrome in the panel. Thus unstained is considered as FMO here. Contour plot, dot plot, bar plot, and histogram showing cell population, percent positive cells and MFI respectively (n=3-6). **(E)** Representative figure depicting back gating used uniformly for all the costimulatory and antigen presentation markers. *p ≤ 0.05, **p ≤ 0.01 and ***p ≤0.001. p-value has been calculated using two tailed paired student's t-test. Data shown in figure is combined from 2-3 independent experiments [A & C]. Error bars represent SEM.
