# Supplementary material for: SMRT and NCoR1 fine-tune inflammatory versus tolerogenic balance in dendritic cells by differentially regulating STAT3 signaling
Source: Front Immunol. 2022 Sep 27;13:910705. doi: 10.3389/fimmu.2022.910705 (PMC9552960; doi:10.3389/fimmu.2022.910705)
Supplement: Supplementary file 8 [file DataSheet_2.pdf]

Supplementary Figure 2

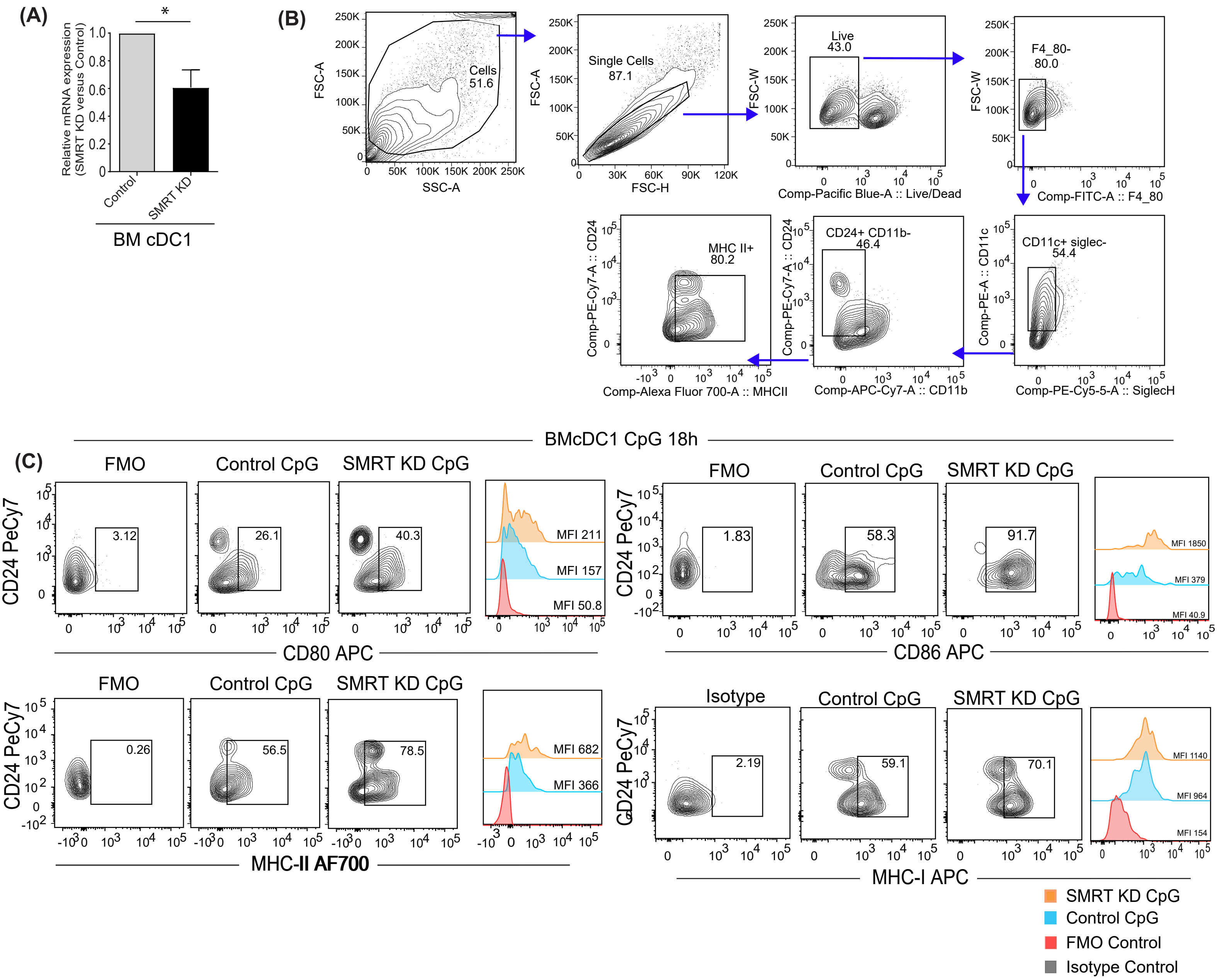

Figure S2. SMRT depleted bone-marrow derived DCs differentiated with FLT3 ligand exhibit enhanced co-stimulation upon CpG stimulation. (A) Relative mRNA expression of *Ncor2* in BMcDC1, error bars represent SEM (n=3). **(B)** Back gating strategy used to identify cDC1 population from FLt3 differentiated murine bone marrow cells. **(C)** Contour plots representing CD80, CD86, MHC-II and MHC-I in 18h CpG stimulated control and SMRT KD BMcDC1.
*p ≤ 0.05, **p ≤ 0.01 and ***p ≤0.001. p-value has been calculated using two tailed unpaired student's t-test. Data shown in figure is combined from atleast 3 independent experiments
