# Supplementary material for: SMRT and NCoR1 fine-tune inflammatory versus tolerogenic balance in dendritic cells by differentially regulating STAT3 signaling
Source: Front Immunol. 2022 Sep 27;13:910705. doi: 10.3389/fimmu.2022.910705 (PMC9552960; doi:10.3389/fimmu.2022.910705)
Supplement: Supplementary file 9 [file DataSheet_3.pdf]

Supplementary Figure 3

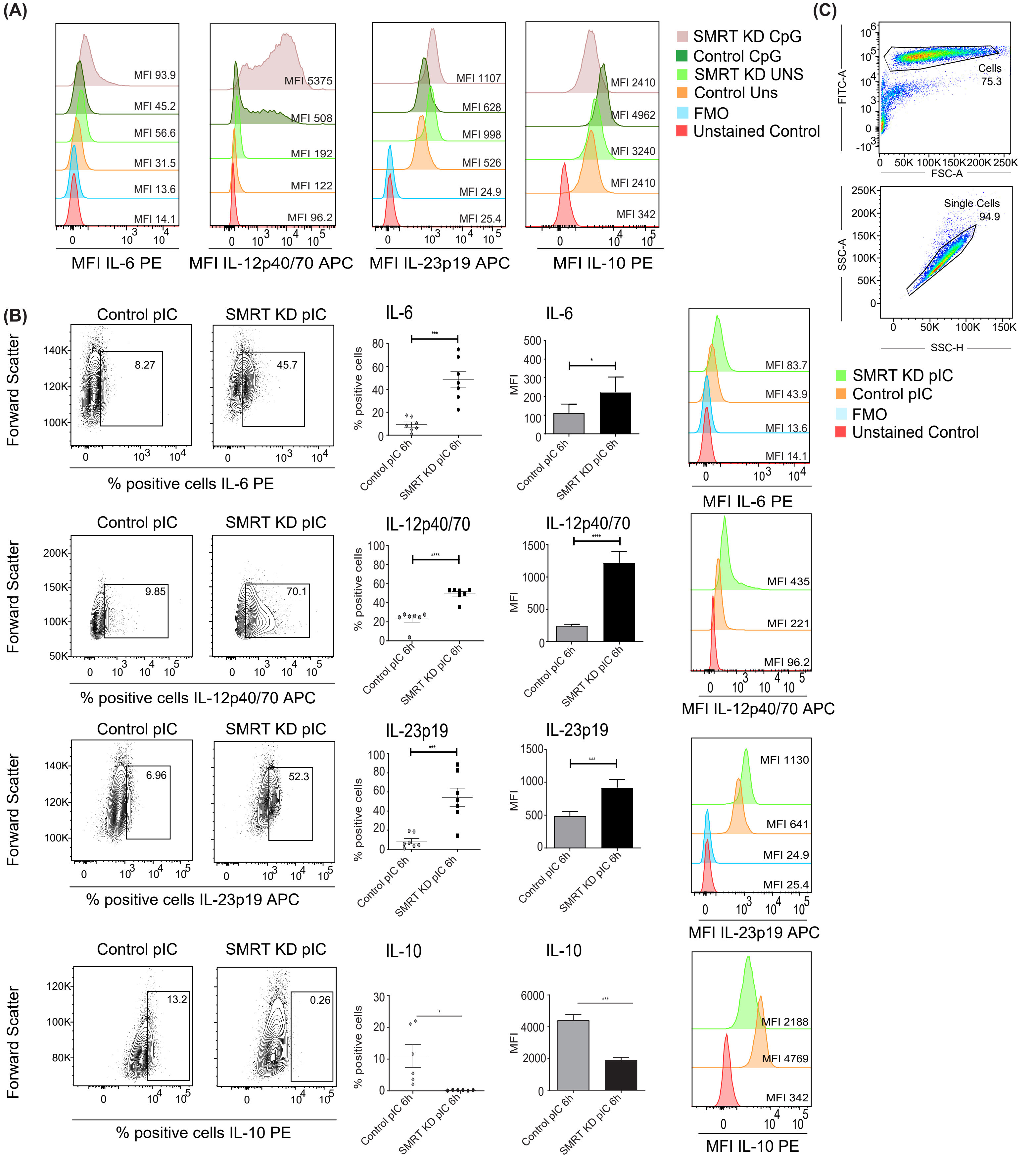

Figure S3. SMRT depleted cDC1 DCs produce enhanced inflammation upon TLR stimulation. (A) Histograms depicting MFI of pro-inflammatory cytokine IL-6, IL-12p40, IL-23p19, and the anti-inflammatory cytokine IL-10 in unstimulated and 6h CpG stimulated control and SMRT KD cDC1 compared to FMO and unstained controls. **(B)** Contour plot, scatter dot plot, bar plot, and histogram showing cell population, percent positive cells and MFI shifts of pro-inflammatory cytokines IL-6, IL-12p40, IL-23p19, and the anti-inflammatory cytokine IL-10 in 6h pIC stimulated control and SMRT KD cDC1 compared to their respective FMO and unstained controls (n=6-7). For staining IL12p40 we did not use any other fluorochrome in the panel. Thus unstained is considered as FMO here. **(C)** Representative figure showing the gating strategy used for all intracellular cytokine analysis. First gate was set on FSC-A and FITC since the cell line has a GFP reporter, and then we discriminated doublets by gating SSC-A and SSC-H.
