# Supplementary material for: SMRT and NCoR1 fine-tune inflammatory versus tolerogenic balance in dendritic cells by differentially regulating STAT3 signaling
Source: Front Immunol. 2022 Sep 27;13:910705. doi: 10.3389/fimmu.2022.910705 (PMC9552960; doi:10.3389/fimmu.2022.910705)
Supplement: Supplementary file 10 [file DataSheet_4.pdf]

Supplementary Figure 4

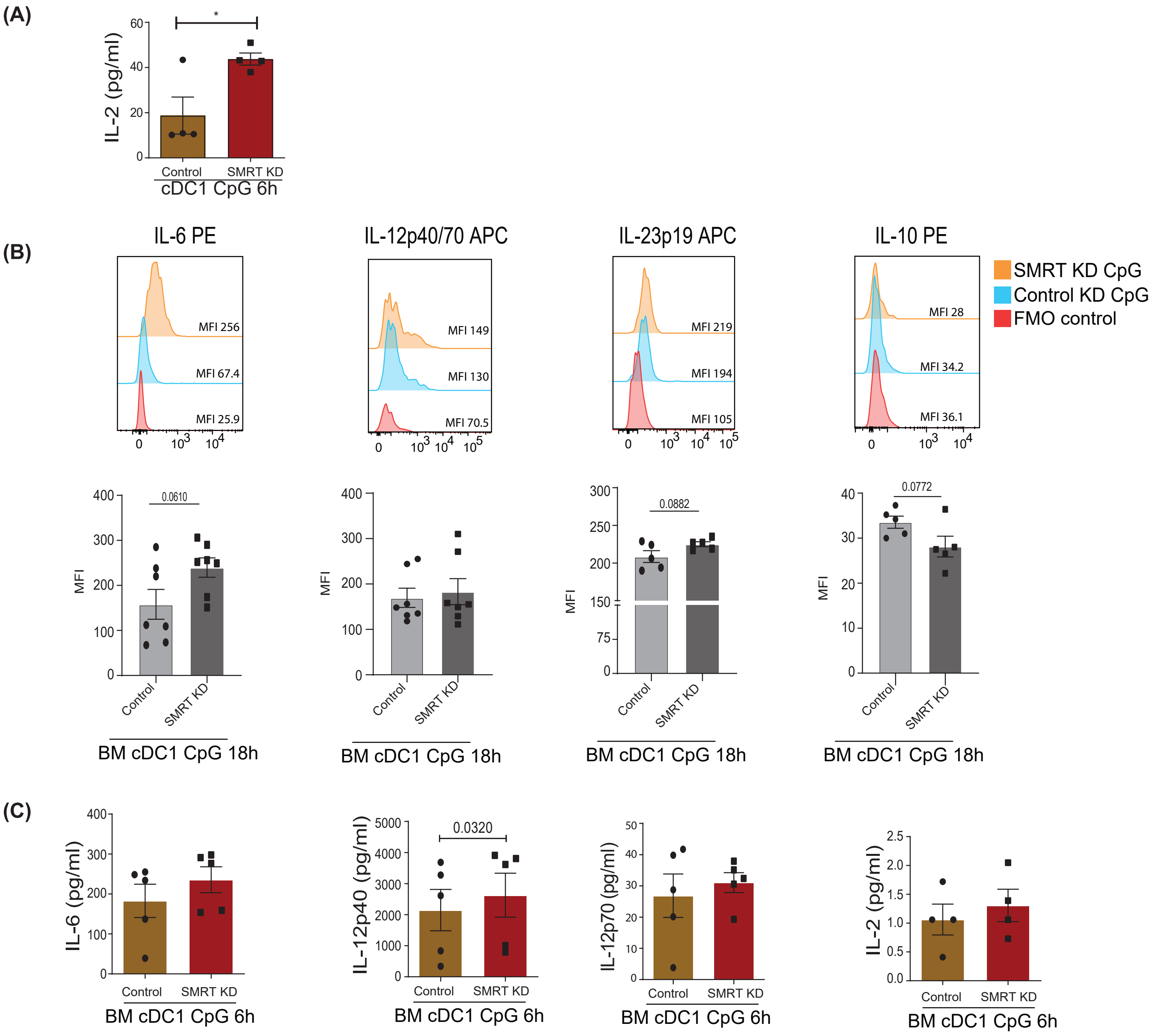

Figure S4. SMRT depleted BMcDC1 exhibit enhanced inflammatory cytokine production upon CpG stimulation. (A) Bioplex cytokine assay showing the quantification of IL-2 cytokine secreted in the culture supernatant of 6h CpG stimulated control and SMRT KD cDC1 (n=4). **(B)** Histogram and MFI scatter plot with bar showing proinflammatory cytokines IL-6, IL-12p40, IL-23p19, and the anti-inflammatory cytokine IL-10 in 18h stimulated control and SMRT KD BMcDC1 (n=5-7). **(C)** Bioplex cytokine assay showing the quantification of IL-6, IL-12p40, IL-12p70, and IL-2 cytokine secreted in the culture supernatant of 6h CpG stimulated control and SMRT KD BMcDC1 (n=4-5).

*p ≤ 0.05, **p ≤ 0.01 and ***p ≤0.001. p-value has been calculated using paired t-test [A and C] and two tailed unpaired student's t-test (B). Data shown in figure is combined from 3 independent experiments. Error bars represent SEM.
