# Supplementary material for: SMRT and NCoR1 fine-tune inflammatory versus tolerogenic balance in dendritic cells by differentially regulating STAT3 signaling
Source: Front Immunol. 2022 Sep 27;13:910705. doi: 10.3389/fimmu.2022.910705 (PMC9552960; doi:10.3389/fimmu.2022.910705)
Supplement: Supplementary file 11 [file DataSheet_5.pdf]

Supplementary Figure 5

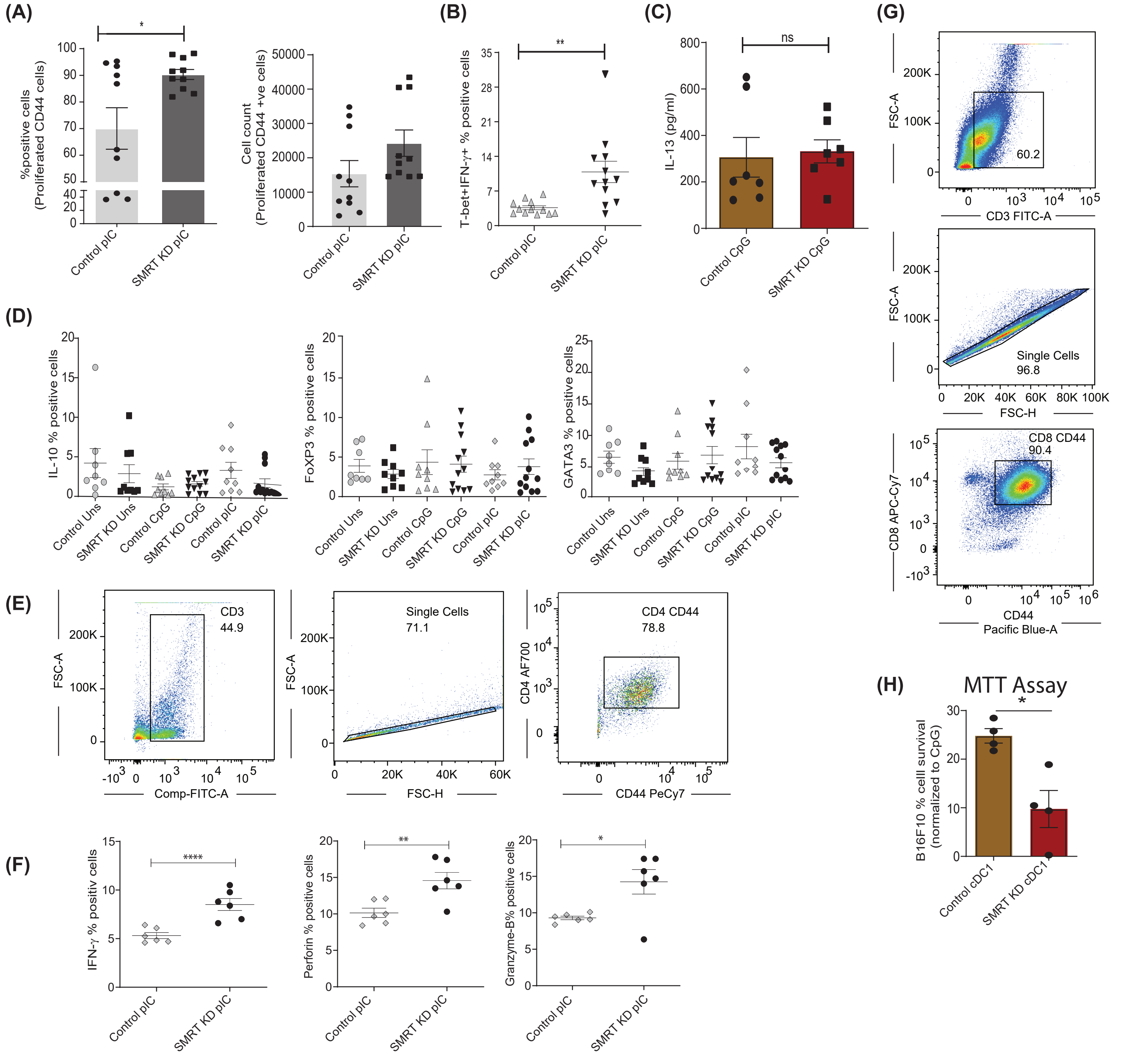

Figure S5. SMRT KD cDC1 increased Th1 and Th17 polarization ex vivo in CD4+ T-lymphocytes and enhanced cytotoxic T-cell activity in CD8+ T-lymphphcytes. (A) Scatter plot with bar depicting proliferated CD44+ OT-II Th-cells co-cultured with control and SMRT KD cDC1 treated with OVA 323-339 peptide overnight followed by pIC stimulation (n=10). **(B)** Dot plots representing co-cultured OT-II Th-cells showing signature transcription factor and cytokine for Th1, T-bet and IFN-γ response in pIC stimulated condition (n=12). **(C)** Bioplex cytokine assay depicting quantification of IL-13 cytokine secreted in the culture supernatant of OT-II Th-cells which were co-cultured with control and SMRT KD DCs pulsed with OT-II peptide and challenged with CpG (n=7). **(D)** Dot plots representing co-cultured OT-II Th-cells showing IL-10, FoxP3, and GATA3 expressing T-cells co-cultured with control and SMRT KD DCs in unstimulated, CpG stimulated, and pIC stimulated condition (n=8-12). **(E)** Representative image showing back gating used for flow cytometry analysis of co-cultured OT-II T-cells. **(F)** Dot plots representing co-cultured OT-I T-cells showing IFN-γ, granzyme-B, and perforin in pIC stimulated condition (n=6). **(G)** Representative figure showing back gating used for flow cytometry analysis of co-cultured OT-I T-cells. **(H)** Scatter plot with bar representing percent cell survival of B16F10 cells loaded with OT-1 T cells co-cultured with control or SMRT KD cDC1 (n=4).

*p ≤ 0.05, **p ≤ 0.01 and ***p ≤0.001. p-value has been calculated using two tailed unpaired student's t-test. Data shown in figure is combined from 3 independent experiments [A], from 4 independent replicates [B], from 3 independent replicates [C], from 2 independent replicates [D], 2 independent replicates [F] and 4 independent replicates [H]. Error bars represent SEM.
