# Supplementary material for: SMRT and NCoR1 fine-tune inflammatory versus tolerogenic balance in dendritic cells by differentially regulating STAT3 signaling
Source: Front Immunol. 2022 Sep 27;13:910705. doi: 10.3389/fimmu.2022.910705 (PMC9552960; doi:10.3389/fimmu.2022.910705)
Supplement: Supplementary file 12 [file DataSheet_6.pdf]

Supplementary Figure 6

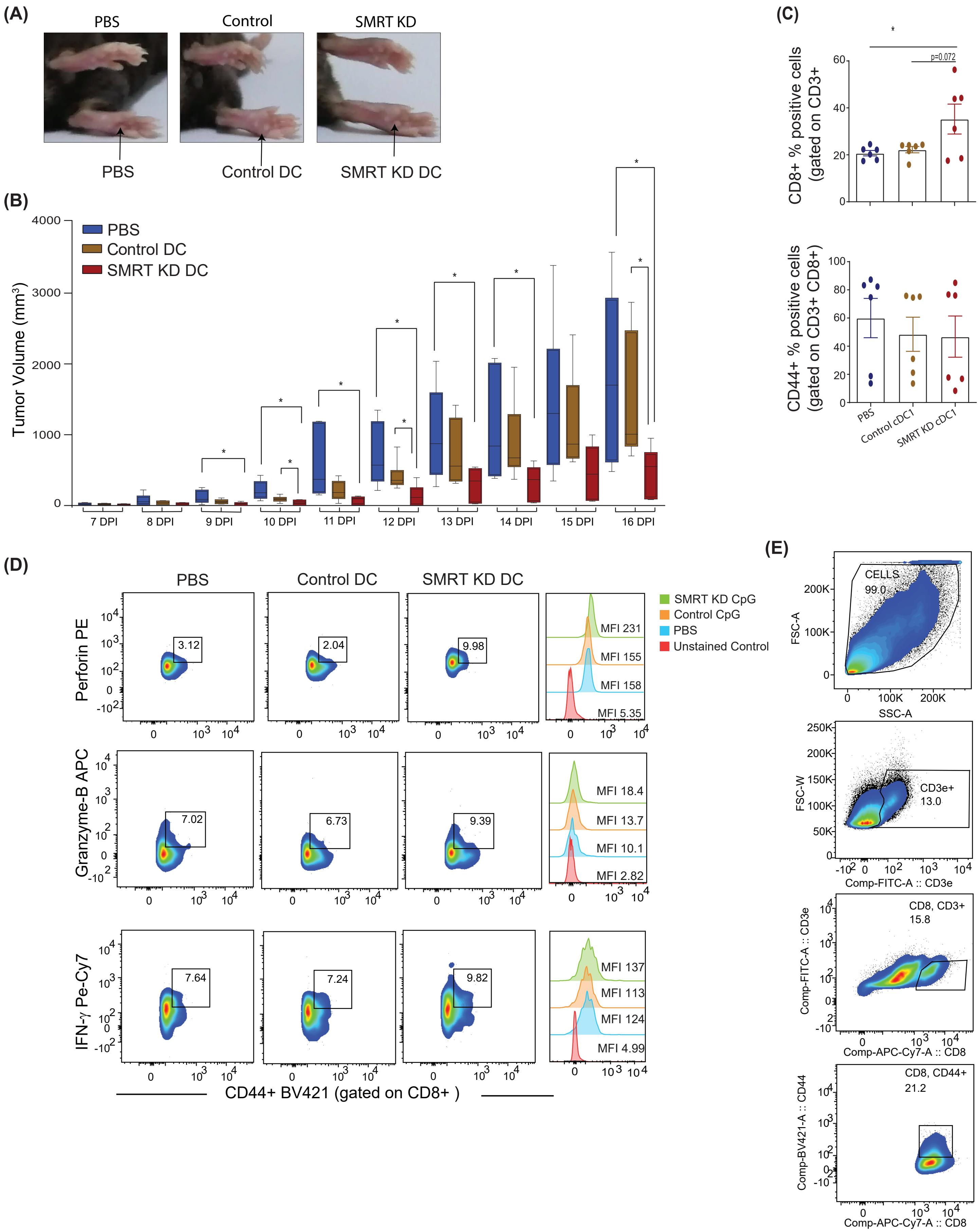

Figure S6. Induction of DTH and B16F10 induced melanoma model in C57BL/6. (A) Image showing foot-pad swelling from PBS, ova pulsed control DCs, and SMRT KD DCs treated mice 72h post ova rechallenge. **(B)** Box plot showing tumor volume that was taken every day starting from 7th day after B16F10 injection till 16 days post tumor rechallenge in PBS, control and SMRT KD DCs injected mice. **(C)** Dot plots depicting percentage of CD8+ and CD44+ T-cells in all three groups of mice (n=6). **(D)** Contour plots and histogram depicting percent positive cells and MFI of perforin, granzyme-B, and IFN-γ in three groups of mice. **(E)** Representataive figure showing back gating used for depicting cytotoxic T-cell. $*p \leq 0.05$, $**p \leq 0.01$ and $***p \leq 0.001$. p-value has been calculated using two tailed unpaired student's t-test. Data shown in figure is combined from 2 independent experiments [A-C]. Error bars represent SEM.
